# Body-World Coupling, Sensorimotor Mechanisms, and the Ontogeny of Social Cognition

**DOI:** 10.3389/fpsyg.2019.03005

**Published:** 2020-01-14

**Authors:** Daniel Rojas-Líbano, Francisco J. Parada

**Affiliations:** Laboratorio de Neurociencia Cognitiva y Social, Facultad de Psicología, Universidad Diego Portales, Santiago, Chile

**Keywords:** sensorimotor mechanisms, social cognition, mechanistic explanation, ontogeny, 4E-cognition, sensorimotor coupling

When closely examined, several biological mechanisms reveal themselves as implementing a physical and dynamical two-way link or coupling between the organism and the world. In these cases, some mechanisms' components can either physically cross the body-world boundary or are brought by the organism's motor actions onto specific sensory surfaces. As with any biological phenomenon, the historical contingencies of these sensorimotor activities generate plastic changes within the organism, that in turn determine its capacities at any given time. Body-world coupling instances are evident in examples that we will describe later, such as breathing, sensori-motor activities, and others. In the present piece, we attempt to position social cognitive phenomena as the result of the mechanisms involved in the organism's coupling history with its world. This coupling constitutes one of the cornerstones of the so called 4E approach to cognition (Newen et al., 2018), from which we will also draw concepts and distinctions in our effort to relate coupling mechanisms with social phenomena. Even though reviewing the 4E approach to cognition escapes the scope of the present piece, we can briefly state that the 4E cognition framework wants to bring multiple approaches together under a sole emblem. It understands cognition as a natural phenomenon, *embodied* in the biophysics of the body which is *embedded* both phylo- and ontogenically into the animal's ecological niche. To the 4E approach, cognition is also *opportunistic* and *promiscuous* as can be *extended* toward the world with objects both material (e.g., technology) and conceptual (e.g., institutions). Finally, the 4E approach thinks cognition as intended for action in an ongoing interactional sense-making process; an *enactive* phenomenon. The 4E cognition framework owes its current form to several landmark work such as the “enactive approach” (Varela et al., 2017), the “distributed cognition branch of cognitive science” (Flor and Hutchins, 1991; Hutchins, 1995), and the “extended mind” proposal (Clark and Chalmers, 1998), among others.

Despite decades of conceptual development of the 4E approach and its diverse subfields, there are many questions regarding its particular implications for neuroscience (e.g., how can neuroscientists can actually implement the 4E approach directly into their research agendas? Is one-person neuroscience necessary?, etc.) (Di Paolo and De Jaegher, 2012; Willems and Francken, 2012). As experimental neuroscientists interested in the interactional nature of cognition, we would like to extract the mechanistic implications of the 4E approach: components, activities, and processes (What?, How?, When?), their context (When?, How?) and their weights (How important?). Epistemologically, we concur with the view that conceives mechanisms as models of the phenomena to explain and consider the building of mechanistic models a fundamental explanatory aim of neuroscience (Craver, 2007). Without a mechanistic picture of the ways in which the 4Es constitute and/or affect cognitive processes, we are left with few tools to further empirical research.

We start by considering relevant distinctions provided by De Jaegher et al. (2010), where *constitutive, enabling*, and *contextual* factors can be identified as the “set of circumstances” which are phenomena themselves. A *contextual* factor modulates the phenomenon, whilst an *enabling* one is necessary for the phenomenon to occur. Finally, *constitutive* factors are processes, parts, and/or pieces that produce the phenomenon itself. What happens if we add a dynamic and mechanistic framework to the De Jaegher, Di Paolo, and Gallagher's proposal? The phenomenon to explain -at any scale (from action potentials to social interaction)- can be understood as the result of the dynamic operation of one or more mechanisms. Such mechanisms comprise components, their activities and the processes in which they participate, whose structural and functional organization in certain conditions produce the phenomenon (Bechtel and Abrahamsen, 2005; Craver, 2007). Thus, we suggest that *constitutive* factors are processes that can be composed of different components of a mechanism under consideration at different moments of time. Examples of components participating in a constitutive fashion are ion channels, for the phenomenon of the action potential, and participating agents for social interaction. In contrast, *contextual* and *enabling* factors are better understood here as elements which interact with mechanisms' components and can change its operation regime. Examples of enabling factors are the existence of ionic gradients across the membrane, for the action potential, and the alertness level of a participant, for social interaction. Examples of contextual factors are, a specific ion channel type for the action potential, and a given environmental setting, for social interaction. It is important to note here that the constitutive, enabling, or contextual quality of a given factor it is not fixed, but can change throughout the organism's ontogeny or history of structural change.

We think our mechanistic view is compatible with the original proposal of De Jaegher et al. (2010). In what follows, we consider the above mentioned points in some detail. We start by examining different mechanisms of body-world coupling, to then propose ways to extend this viewpoint into social-cognitive phenomena, considering the organism's ontogeny.

## Body-World Coupling

### Active Coupling Through Sensorimotor Activities

An example of body-world coupling is represented by an animal's sensory-motor activities. In situations where the sensory processes are important for the organism, there is usually a profound interplay between the animal's actions and the operation of its sensors (Rojas-Líbano et al., 2014). This is evident in motor actions associated with sensory sampling of the environment: touching, sniffing, echolocating, whisking, visual scanning. These actions allow the animal to bring stimuli to sensory surfaces. In most of these cases, stimuli sampling takes place in the wider context of adaptive and context-sensitive behavior. The animal actively moves its sensory systems to make decisions about navigation, small displacements, further explorations, language actions, and the like (Ganguly and Kleinfeld, 2004; Hayhoe and Ballard, 2005; Rojas-Líbano and Kay, 2012; Clark, 2013; Arce-McShane et al., 2016).

**Figure 1:**
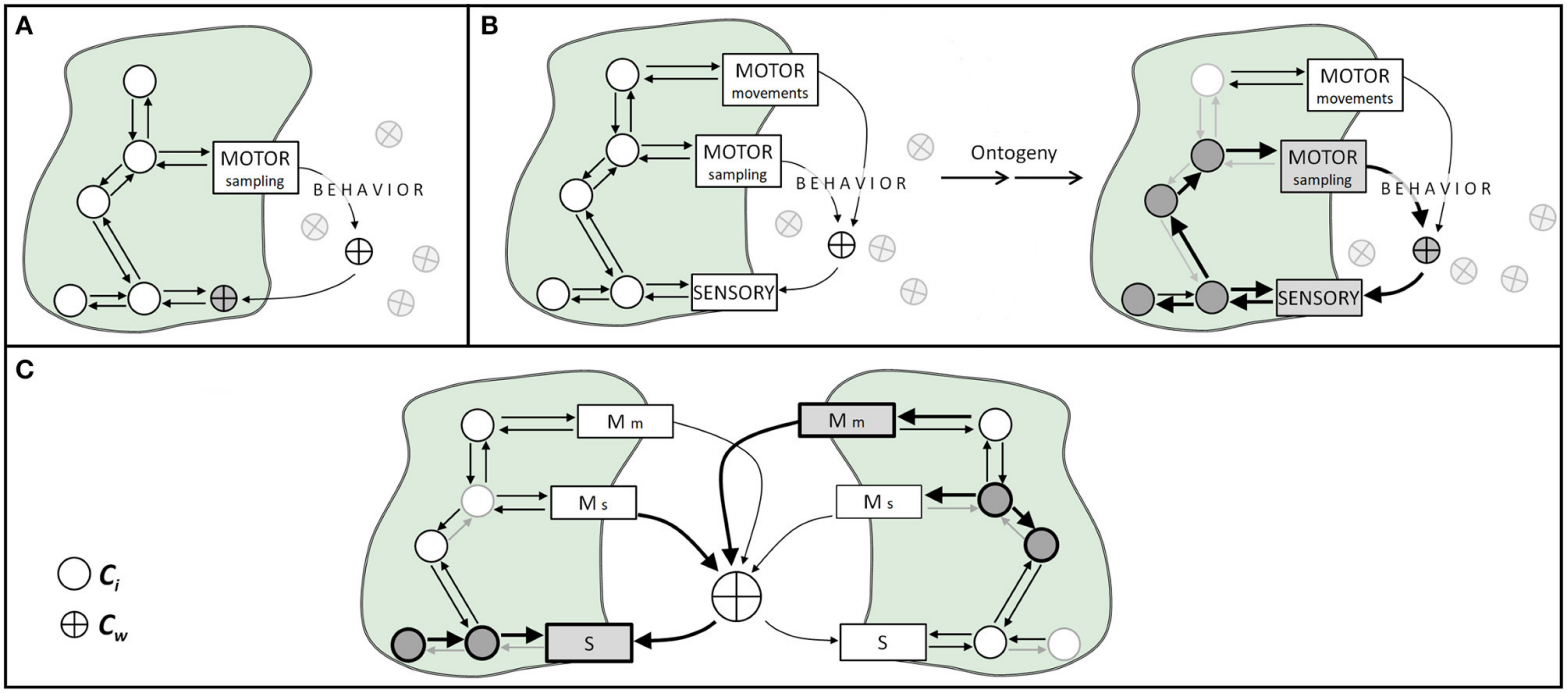
Illustration of body-world coupling, sensorimotor mechanisms, and the ontogeny of social cognition. Circles represent mechanisms' components internal to the organism (*C*_*i*_), and crossed circles depict world components (*C*_*w*_). Arrows represent causal effects between components. **(A)** In non-sensorimotor body-world couplings, an organism's motor activities capture world's components and makes them interact with body components. **(B)** In sensorimotor body-world couplings, through active motor sampling activities (e.g., sniffing, touching, fixating) the organism dynamically brings world components onto sensory surfaces. During ontogeny, the occurrence (or not) of specific sensorimotor activities produces plastic changes in body components, represented here as weighted arrows and circles. **(C)** Social cognition as a process grounded in sensorimotor coupling. World components relevant to sensorimotor coupling could well be stimuli produced by another organism, such as physical stimuli resulting from communication processes, and different agents coupled with shared world components can lead to social cognitive phenomena. This kind of sensorimotor coupling might entail different plastic processes within each organism (represented as different weighted arrows inside each agent). S, sensory; Mm, motor actions for movement; Ms, motor actions for sampling.

The appropriate interplay or coordination between motor actions and sensory activations requires the participation of certain components of the world in the sensory-motor mechanism. Therefore, cognitive activities involving any type of movement will demand some environmental components to become participants of a mechanism (i.e., a transiently constitutive factor). If we manipulate world conditions that interfere with this loop, we can potentially destroy the organism's coupling in the sense that we decrease its ability to interact coherently with its world. Examples are everywhere. Sniffs manipulate the number of odor molecules drawn onto the olfactory epithelium, as well as the rate (i.e., flow) at which those molecules travel through the nose (Rojas-Líbano and Kay, 2012). Tactile (e.g., whisker, finger) movements are coordinated with body movements and control the spatiotemporal frequency at which mechanical stimuli contact the skin cutaneous receptors (Kurnikova et al., 2017). Eye/head/body movements effectively displace the photoreceptor surface so as to receive photons coming from specific objects from the visual scene (Schroeder et al., 2010), and mechanisms such as the accommodation reflex modify the amount and direction of light that reaches the retina, via the modification of pupil size and lens width (Michael-Titus et al., 2010). All these motor activities *manipulate* world components and -through this manipulation- cause changes onto sensory surfaces (Figures 1A,B). Thus, world components continuously move back and forth from participating in processes contextual or enabling to constitutive factors for a given point in time and a given sensorimotor act.

### Other Examples of Coupling

Some mechanisms are part of the basic autonomy of a living being and can be independent of active volitional control. There are many examples, such as coupling through circadian rhythms or, at the cellular scale, through membrane potential maintenance, nutrient exchange, and structural interactions with the extracellular matrix. However, for the sake of simplicity, let us specifically focus on mammalian breathing as a non-sensorimotor example of a mechanism that allows an organism to functionally couple with its world. We know a fairly good deal of the neural mechanisms that implement breathing in mammals (Feldman and Del Negro, 2006). In this process, the animal actively exchanges components with its world, specifically air volumes with different amounts of oxygen and carbon dioxide. Neurons in the brainstem periodically fire impulses that eventually send activity down the phrenic nerve, delivering acetylcholine onto the muscle cells of the diaphragm. The diaphragm then contracts, expanding the thoracic cavity and increasing lung volume. This expansion draws air from the organism's surroundings into the lungs. Finally, the diaphragm relaxes, pushing air from inside the lungs back to the exterior of the animal's body. Accompanying the volume exchange there is a substance exchange: inspired air is more enriched in oxygen than expired air, which in turn is more enriched in carbon dioxide. At a molecular scale, we can conceive the mechanism as a continuous exchange of molecules. From an outside reservoir enriched in oxygen molecules, the organism draws oxygen inside and pushes out carbon dioxide. This mechanism operates as long as the animal preserves its biological autonomy.

Now, consider what happens when we intervene on the external side. Lowering the air oxygen concentration causes a decrease in blood oxygen, which in turn activates peripheral and central chemoreceptor neurons (Teppema and Dahan, 2010). The activation of the latter triggers an increase in drive to the diaphragm, resulting in stronger, and more frequent breathing cycles. Something similar happens if we prevent molecules from crossing the boundary, say by occluding the airway. This indicates that by manipulating the external state of affairs, and/or by preventing physical exchanges across the body-world boundary, we causally intervene in the mechanism. We propose that this is a feature of mechanisms that couple body and world. It is also trivially true that several manipulations of the external conditions can causally affect the body, such as when the body is hit, for example, by a heavy object. But in those cases the world component involved was not implicated in a regular mechanism with the organism.

## Ontogeny, Social Cognition, and Body-World Coupling Mechanisms

In the cases described above, and in many others, what we see is a physiological mechanism that contains -as part of its regular components- some element(s) of the world. By altering either internal or external components, we alter the mechanism operation (Figures 1A,B).

Let ***M*** be a (neuro)physiological mechanism (e.g., respiration, sensorimotor operations, circadian rhythms) containing internal components ***Ci*** which normally interact with some world's components ***Cw*** (any processes and/or entities, whether living or not, present outside the organism's physical body). Traditionally, it is conceived that the operation of ***M*** depends on ***Ci*** alone. However, for relevant biological phenomena, such as respiration or sensorimotor activities, ***Cw*** are mechanism components, participating in the resulting processes, and therefore we think is useful to regard them as constitutive^1^. Likewise, other ***Cw*** would be enabling and/or contextual, depending on the phenomena under consideration. Considering ***Cw*** as constitutive and/or enabling elements of a given ***M***, we can further state that many organizational principles of the brain -generated from multiple operating mechanisms- will be much better explained by incorporating their relationship to the world (Clark and Chalmers, 1998; Cosmelli and Thompson, 2010; Parada and Rossi, 2018).

We could also say that the operation of a given ***M*** will depend on the organism's past and current temporospatial contingencies (i.e., both ***Ci*** and ***Cw***). A key notion here is that biological mechanisms are not timeless laws, but *historically contingent processes* (Craver, 2007). Consider, as an example, the mechanisms of neural plasticity. It has been shown that present neuronal properties -both structural and functional^2^- are dependent on the neuron's previous interactions with its immediate environment (Rose and Rankin, 2001; Bailey et al., 2015; Andersen et al., 2017; Schulz and Lane, 2017). Importantly, this is not a special feature of neurons, but a general biological phenomenon. The actual state and capacities of any organism are activity- and ontogeny-dependent, and are always intertwined with the environment in which ontogeny takes place (Stagg et al., 2011; Kelly et al., 2012; Ganguly and Poo, 2013; Sale et al., 2014; Fields, 2015). Social-cognitive phenomena can be conceived, within this framework, as interactions occurring through the sharing of some ***Cw*** between the agents engaged in it (Figure 1C).

Taking into account the dependence on history of biological mechanisms, it is particularly relevant to distinguish the role of ***Cw*** at different moments along ontogeny. At different moments, the weight of a ***Cw*** could play a role as a *constitutive, enabling*, or *contextual* factors in a given phenomenon. For example, the case of behavioral habituation shows that, under sustained interactions, responses to the same ***Cw*** can decrease drastically, turning a ***Cw*** stimulus from a once-constitutive element to a mere contextual perturbation (Brunelli et al., 1976). In what follows, we use these ideas to propose a link between ontogenic mechanisms of body-world coupling and social interactions.

Social interaction starts very early during development, from prenatal experiences to turn-taking in babies to early verbalizations in infants (Siddiqui and Hägglöf, 2000; Kugiumutzakis, 2017; Quigley et al., 2017). From the point of view of mechanisms of body-world coupling, these developmental changes correspond to an increment in the allowed complexity of sensorimotor interactions. Mechanistically, increased sensorimotor complexity can be reached by reducing the sensorimotor contingencies' dimensionality, using both history of interactions and sensorimotor function. This is the organism's current morphological shape, as a product of previous body-world couplings in time, affords more complex actions contained in appropriate ecological niches. A now-classic example is the theoretical (Smith et al., 1999) and empirical (Smith and Thelen, 2003) dynamical systems account of the A-not-B error in infants (Piaget, 1962). Briefly, the processes underlying the perseverative reaching seen in the A-not-B error are not only continuously tied to the infant's sensorimotor system but also to her history of interactions (Spencer et al., 2011). From our perspective, evidence from animal models suggests a constitutive role of external factors such as maternal state during gestation (Kofman, 2002), maternal care/physical contact (Cancedda et al., 2004; Sale et al., 2004), as well as overall environmental conditions (Cai et al., 2009). Similar effects have been reported in humans; social, cultural, and/or physical environmental conditions in earlier developmental stages might bias -or even shape- bio-psycho-social trajectories (Guzzetta et al., 2009; Bowers and Yehuda, 2016; McEwen, 2017). Later in life, most of these factors can become enabling and/or contextual.

A more speculative example -directly related to social cognition- could be found in language; a higher-level cognitive phenomenon profoundly sensitive to ontogenic changes (Peña et al., 2003; Dehaene-Lambertz et al., 2008; Mampe et al., 2009; Mahmoudzadeh et al., 2013; Werker and Gervain, 2013; Werker and Hensch, 2015). The available evidence indicates that human auditory learning starts in the third trimester of gestation (Shahidullah and Hepper, 1994; Hepper, 1996). We further interpret this evidence as suggesting a constitutive role for prenatal listening experiences (***Cw***) in the specific physiological and developmental trajectory that gives rise to speech processing brain structures (***Ci***) (Wermke and Friederici, 2004). Between the 8th and 10th month of age, this body-world coupling begins its consolidation, allowing infants to extract statistical regularities (Saffran et al., 1996), which we conceive as a dimensionality reduction of the complex linguistic world (Werker and Tees, 1984; Maurer and Werker, 2014)^3^. Following our interpretation of these data, listening experiences and verbal interactions (***Cw***) become contextual factors after the 10th month of age (Werker and Curtin, 2005; Werker and Hensch, 2015). We further speculate that such change, from constitutive to contextual, illustrates the dimensionality reduction required for the appearance of more complex sensorimotor operations, such as actively seeking learning opportunities, maximizing informative interactions, and the beginning of adult-like social interactions (Begus et al., 2016). We still lack both data and tools to appropriately model the role, weight, and influence of external factors (from physical interplay to social interactions to processes unfolding from them) in the emergence of social-cognitive functioning and the overall biophysics of human experience.

## Closing Remarks

The present opinion piece seeks to facilitate a mechanistic approximation to multi-level phenomena, grounding social cognition, and social interaction into time-dependent functional and structural components and their interplay; a goal for the 4E approach to cognition. Furthermore, it points to the need of modeling, through experimental manipulations, the weight and influence of both internal [i.e., (neuro)physiological] and external (i.e., objects, processes, other people) components at a given developmental period. This modeling can be achieved through tools derived from network science and/or machine learning techniques (Vespignani, 2011; Boonstra et al., 2015; Sekara et al., 2016; Shine et al., 2016; Avena-Koenigsberger et al., 2017; Aguilera, 2018; Parada and Rossi, 2018). Furthermore, implementing scalable experimental paradigms (Parada, 2018; Matusz et al., 2019; Shamay-Tsoory and Mendelsohn, 2019) and generating novel hypotheses of interacting brain/body systems functioning during natural cognition (De Jaegher et al., 2010, 2016; Di Paolo and De Jaegher, 2012; Gramann et al., 2014; Ladouce et al., 2017; Parada, 2018; Parada and Rossi, 2018) are among the most outstanding challenges for the 4E-cognition research program. We believe that the incorporation of a mechanistic framework facilitates meeting those challenges and advancing a deeper understanding of cognitive phenomena, social, and otherwise.

## Author Contributions

DR-L and FP conceptualized the present work and wrote the current version for publication.

### Conflict of Interest

The authors declare that the research was conducted in the absence of any commercial or financial relationships that could be construed as a potential conflict of interest.

## Footnotes

^1^We follow Craver (2007) in using manipulability as a criterion for recognizing mechanisms' components. Briefly stated, if interventions on the mechanism as a whole are accompanied by changes of a potential component, and if interventions on the component produce, in turn, changes in the mechanism, then the component under consideration is a mechanism's component.

^2^And hence of the networks in which the neuron participates.

^3^This is also seen in other aspects of perceptual development (Scott et al., 2007).
